# Contemporary use of P2Y_12_ inhibitors in patients with ST-segment elevation myocardial infarction referred to primary percutaneous coronary interventions in Poland: Data from ORPKI national registry

**DOI:** 10.1007/s11239-017-1579-9

**Published:** 2017-10-26

**Authors:** Tomasz Rakowski, Zbigniew Siudak, Artur Dziewierz, Krzysztof Plens, Paweł Kleczyński, Dariusz Dudek

**Affiliations:** 1 2nd Department of Cardiology, Institute of Cardiology, Jagiellonian University Medical College, Kopernika 17 Street, 31-501 Krakow, Poland; 2 Department of Interventional Cardiology, Institute of Cardiology, Jagiellonian University Medical College, Krakow, Poland; 3 KCRI, Krakow, Poland

**Keywords:** Myocardial infarction, Primary PCI, Antiplatelet therapy, Registries

## Abstract

According to guidelines, it is recommended to give P2Y_12_ inhibitors (preferably ticagrelor or prasugrel) at the time of first medical contact in patients with STEMI. However, in real life antiplatelet treatment strategies are different among countries. We analyzed data on antiplatelet treatment in STEMI patients included into Polish ORPKI national registry. A total of 23,139 STEMI patients from 153 invasive cardiology centers were reported in ORPKI registry between September 2015 and August 2016. Finally 19,437 patients from 122 centers (immediate PCI in 94%) were included into the analysis (lack of ticagrelor or prasugrel usage reported in 31 centers). The dominant P2Y_12_ inhibitor was clopidogrel (69%) with a high rate of precathlab administration (51.3%). Ticagrelor was administered in 10.1% of patients (2.3% during precathlab phase) and prasugrel in 1.1% (0.4% precathlab). The periprocedural switch from clopidogrel to newer generation oral P2Y_12_ inhibitors was rare (to ticagrelor: 2%; to prasugrel: 0.15%). Analysis of data from top 10 centers with the highest rate of newer generation P2Y_12_ inhibitors usage (1295 patients) revealed ticagrelor administration in 43.1% (prasugrel in 3%). During precathlab phase higher proportion of ticagrelor instead of clopidogrel (ticagrelor 17.9%, clopidogrel 29.8%) and higher rate of periprocedural switch from clopidogrel to ticagrelor (11.9%) was found comparing to all centers data (p < 0.001 for all). The strategy of precathlab administration of P2Y_12_ inhibitors applies to about half of STEMI patients in Poland. Generally, ticagrelor or prasugrel use is low, and not equally distributed among centers. In centers with high usage, ticagrelor is main newer generation P2Y_12_ inhibitor for precathlab and periprocedural administration.

## Introduction

Antiplatelet therapy plays a key role in the treatment of patients with ST-segment elevation acute myocardial infarction (STEMI) referred to primary percutaneous coronary intervention (PCI). According to current guidelines, prasugrel and ticagrelor are the first choice antiplatelet drugs in patients with STEMI [1]. However, practical approach to antiplatelet treatment is quite heterogeneous among primary-PCI networks worldwide [2–7]. Generally, in daily clinical practice, the usage of those drugs as compared to clopidogrel is lower than expected. The timing of P2Y_12_ administration (precathlab vs. periprocedural) is also a matter of debate due to lack of clear evidence from clinical trials on advantage of any strategy [8, 9]. Practical approach varies from network to network and include early prehospital but also periprocedural administration [10]. Moreover, the switch from and to clopidogrel is quite frequent both during in-hospital stay and follow-up [11]. Thus, we sought to analyze data on antiplatelet treatment strategies in STEMI patients in Poland based on patients included in large scale Polish ORPKI national registry.

## Methods

Presented analysis is based on data stored in the electronic database of National PCI Registry (ORPKI) operated by the Jagiellonian University Medical College in Krakow. ORPKI is a national registry collecting data on all percutaneous procedures in interventional cardiology performed in Poland [7]. For the study data on patients with STEMI included into the registry between September 2015 and August 2016 were analyzed. Centers using only clopidogrel (no ticagrelor, no prasugrel) were excluded. All procedures were carried out according to current medical standards. The decision to use P2Y_12_ inhibitor (type, time, dose) was the operator’s choice according to clinical practice standards.

Due to relatively low penetration of newer generation oral P2Y_12_ inhibitors in the studied cohort, we decided to perform additional analysis based on data from top 10 centers with the highest rate of ticagrelor/prasugrel usage to better understand the characteristics of the population treated with those drugs. The minimal number of patients for the center to be included in top 10 group was 50 per center in the above mentioned period.

### Statistical analysis

The analysis was performed based on standard descriptive statistics. Quantitative variables were presented as median with interquartile range (IQR) and categorical variables as percentages. Continuous variables were expressed as mean ± standard deviation (SD) or median and IQR. Normality was assessed by the Shapiro–Wilk test. Equality of variances was assessed using the Levene’s test. Differences between groups were compared using the Student’s or the Welch’s t-test depending on the equality of variances for normally distributed variables. The Mann–Whitney U test was used for non-normally distributed continuous variables or for ordinal variables. Categorical variables were compared by the Fisher’s exact test for 2 × 2 tables or by Pearson’s chi-squared test for contingency tables with higher dimensions. The level of statistical significance was set at p < 0.05. All analyses were carried out with JMP^®^, Version 12.2.0 (SAS Institute Inc., Cary, NC, USA).

## Results

### Study population

A total of 23,139 STEMI patients from 153 invasive cardiology centers in Poland were reported in the ORPKI registry from September 2015 to August 2016. Finally, 19,437 patients from 122 centers were included into the analysis (lack of ticagrelor and prasugrel usage was reported in remaining 31 centers). Study group represents real-life STEMI population (Table 1). Median time from pain onset to first balloon inflation was 248 (Q1–Q3: 146–540) min. The radial approach was selected for 72% of patients. At baseline, patent (TIMI grade 2/3 flow) infarct-related artery (IRA) was found in 27%. The left anterior descending coronary artery was identified as IRA in 41% of patients. Immediate PCI was performed in 93.7% with aspiration thrombectomy in 13% and stent implantation in 92.4% (95% DES). Final TIMI 3 flow grade after PCI was presented in 91.4%. Periprocedural anticoagulant was unfractionated heparin in most of the cases (including pracathlab administration in 47.7%) except low molecular heparin in 4.4% and bivalirudin in 0.3% of patients.

**Table 1:** Characteristics of total study cohort

Variable		n = 19,437
Age (years)	Me(Q1;Q3)	64.00 (57.00;74.00)
Gender (male)		67.7%
Diabetes mellitus		18.1%
Previous stroke		3.3%
Previous myocardial infarction		13.6%
Previous PCI		12.5%
Previous CABG		2%
Smoking		29%
Arterial hypertension		60.4%
Chronic renal disease		3.5%
COPD		2.2%
Precathlab cardiac arrest		5.5%
Killip class on admission	1	83%
	2	9.5%
	3	3.4%
	4	4.1%
Vascular access	Left radial	14.7%
	Right radial	57.2%
	Femoral	27.5%
	Other	0.6%
Immediate PCI		93.7%
TIMI before PCI	0	59.6%
	1	13.3%
	2	14.0%
	3	13.1%
TIMI after PCI	0	2.6%
	1	1.6%
	2	4.4%
	3	91.4%

*CABG* coronary artery bypass graft, *COPD* chronic obstructive pulmonary disease, *PCI* percutaneous coronary intervention, *TIMI* thrombolysis in myocardial infarction

### Antiplatelet treatment

#### Total study cohort

**Fig. 1:**
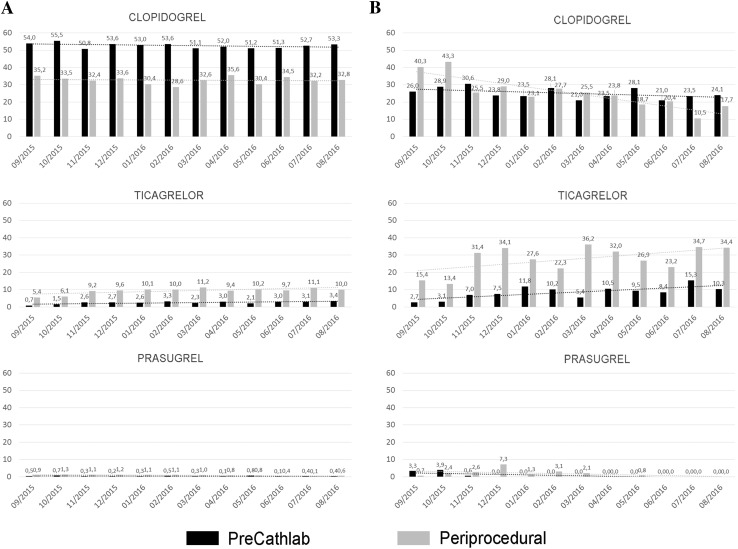
The distribution of clopidogrel, ticagrelor and prasugrel for both precathlab and periprocedural administration. **A** Data from all centers, **B** data from top 10 centers (see text for details)

Aspirin was given before cathlab in 72%, and during the procedure in 25% of patients. The dominant P2Y_12_ inhibitor was clopidogrel (69%) with a high rate of precathlab administration (51.3% of total cohort). Ticagrelor was administered in 10.1% of patients (2.3% during precathlab phase) and prasugrel in 1.1% (0.4% precathlab). The periprocedural switch from clopidogrel to newer generation oral P2Y_12_ inhibitors was rare (to ticagrelor: 2%; to prasugrel: 0.15%). The distribution of clopidogrel, ticagrelor and prasugrel for both precathlab and periprocedural administration is presented in Fig. 1A. Glycoprotein IIb/IIIa inhibitors were administered in about one-third of patients (mostly eptifibatide).

#### Analysis based on top 10 centers with the highest rate of ticagrelor or prasugrel usage

When analyzed data from top 10 centers with the highest rate of ticagrelor or prasugrel usage (1295 patients; clopidogrel 53.9%; ticagrelor 43.1%; prasugrel 3%) we found relatively higher proportion of ticagrelor instead of clopidogrel during precathlab phase (ticagrelor 17.9%, clopidogrel 29.8%; p < 0.001 comparing to all centers data for both) and higher rate of periprocedural switch from clopidogrel to ticagrelor (11.9%; p < 0.001 comparing to all centers data). The distribution of clopidogrel, ticagrelor, and prasugrel are presented in Fig. 1B. Patients treated with ticagrelor or prasugrel comparing to clopidogrel were younger, more often male with a lower rate of chronic obstructive pulmonary disease (COPD). A higher rate of TIMI 3 flow grade after PCI was found in ticagrelor or prasugrel patients comparing to the clopidogrel group (Table 2). All centers included in Top 10 were high volume with more than 400 PCI/year (from about 600 to about 2500 PCI/year).

**Table 2:** Characteristics of patients from top 10 centers with the highest rate of newer generation P2Y_12_ inhibitors usage

Variable		Prasugrel/ticagrelor (n = 597)	Clopidogrel (n = 698)	p
Age (years)	Me(Q1;Q3)	63.00 (55.00;71.00)	66.00 (58.00;77.00)	< 0.0001
Gender (male)		74.2%	66.2%	0.0017
Diabetes		16.6%	20.6%	0.06
Previous stroke		2.7%	3.7%	0.29
Previous myocardial infarction		16.8%	19.3%	0.23
Previous PCI		16.8%	19.3%	0.24
Previous CABG		1.8%	2.3%	0.57
Smoking		33.5%	33%	0.83
Arterial hypertension		72%	68.9%	0.22
Chronic renal disease		3.5%	4.4%	0.40
COPD		0.8%	2.7%	0.0122
Killip class on admission	1	87.8%	83.3%	0.19
	2	6%	8.7%	
	3	2.4%	3.1%	
	4	3.8%	4.9%	
Vascular access	Radial left	6.1%	8%	0.007
	Radial right	58%	48.3%	
	femoral	35.5%	43.3%	
	Other	0.4%	0.4%	
TIMI before PCI	0	64.7%	61.4%	0.22
	1	13.2%	15.2%	
	2	12.3%	11.1%	
	3	9.8%	12.3%	
TIMI after PCI	0	1.2%	3.2%	0.0047
	1	0.7%	1.1%	
	2	3%	4.8%	
	3	95.1%	91.9%	
LAD treated during PCI		39.8%	38.1%	0.54

*CABG* coronary artery bypass graft, *COPD* chronic obstructive pulmonary disease, *LAD* left anterior coronary artery, *PCI* percutaneous coronary intervention, *TIMI* thrombolysis in myocardial infarction;

## Discussion

The main findings of our study are as follows: the usage rate of newer generation P2Y_12_ inhibitors in STEMI patients referred to primary PCI in Poland is still low. In addition, there are significant differences between networks in the proportion of ticagrelor/prasugrel and clopidogrel (from zero to almost half of patients) use. The main newer generation P2Y_12_ inhibitor is ticagrelor, the rate of periprocedural escalate switching (from clopidogrel to ticagrelor/prasugrel) is up to 12% in top newer generation P2Y_12_ inhibitor centers, the precathlab administration of P2Y_12_ inhibitors is relatively high but heterogeneous in terms of clopidogrel to ticagrelor/prasugrel proportions.

According to guidelines, newer generation P2Y_12_ inhibitors (ticagrelor and prasugrel) are preferred over clopidogrel in patients with STEMI [1]. However, many reports shows that clopidogrel is still frequently used despite lack of contraindications to ticagrelor and prasugrel. In the GRAPE Study (data based on year 2012 registry; acute coronary syndromes (ACS) patients with 53% of STEMI), the initial choice of clopidogrel was present in about 70% of patients. However, at discharged this number was significantly lower (less than 40%) what may suggest a conservative approach in the acute phase (clopidogrel) following in-hospital treatment escalate to newer generation oral P2Y_12_ inhibitors [2]. Those proportions are different in recently published data. In the rapport from four centers from Austria (data from year 2015) clopidogrel was administered initially in 29% of patients with ACS (22% of STEMI patients) and 27% at discharge (STEMI 18.7%), confirming that majority of patients were treated with newer generation P2Y_12_ inhibitors [3]. In EYESHOT Study from Italy (data collected in years 2013/2014) clopidogrel at discharge was prescribed in more than half of patients with ACS (32% in STEMI patients treated with primary PCI) [4]. In APATHY Registry (also data from Italy, year 2014) clopidogrel was administered in 52% of patients [5]. The report from the US based registry on ACS patients enrolled from 2011 to 2014 have shown clopidogrel usage in 77.6%. However, the penetration of newer generation P2Y_12_ inhibitors was higher in STEMI (clopidogrel usage in less than 60% of patients) [6]. In our report (data from years 2015/2016) the rate of ticagrelor and prasugrel usage was low and there were large differences between centers in Poland (from zero to almost half of STEMI patients). It should be underlined that newer generation P2Y_12_ inhibitors are not reimbursed in Poland. Low prasugrel availability on Polish market may explain a large discrepancy between ticagrelor and prasugrel usage. Moreover, the reason for high usage of clopidogrel is not clear and the presence of contraindications may not explain it. In above-mentioned registry from Austria, about 55% of patients discharged on clopidogrel had no absolute contraindications to newer generation P2Y_12_ inhibitors [3]. In GRAPE registry, the selection of clopidogrel at discharge was less preferred in about 75% of patients [2]. In APATHY Registry only 48% of patients received antiplatelet therapy according to guidelines [5]. Data from registries showed that “typical” patient for newer generation P2Y_12_ inhibitors administration is relatively young male, undergone PCI for STEMI and without COPD and without a need for oral anticoagulation, with low bleeding risk. In our study, it was not possible to analyze all factors to assess current adherence to guidelines but we also observed that newer generation P2Y_12_ inhibitors were more often administered to young male patients with a lower rate of COPD. During hospitalization, the rate of switching from and to clopidogrel was different in different reports. In GRAPE registry one-third of patients initially treated with clopidogrel was switched to ticagrelor or prasugrel during hospitalization [2]. On the contrary, in EYESHOT Registry the escalate switch occurred only in 3.6% during the procedure and 14.2% of patients at discharge among patients receiving revascularization [4]. Similarly, Tscharre et al. showed a low rate of in-hospital switching between clopidogrel and newer generation P2Y_12_ inhibitors [3]. The SCOPE Registry was focused on the incidence of oral P2Y_12_ inhibitors switching in ACS patients treated with PCI and on the 30-day outcomes. About 40% of patients were initially treated with clopidogrel. The switching rate was 9.6% (2.3% in cathlab, 3.3% at discharge, and 5.1% at follow-up). The de-escalate switching (from newer generation to old P2Y_12_ inhibitors) in an early phase of ACS was an independent predictor of net adverse events [12]. On the other hand results of recently published TOPIC Trial suggests that the “late” switch to clopidogrel (after 1 month from PCI in ACS) may reduce the rate of bleeding events without increasing the risk of ischemic events [13]. In TROPICAL-ACS Trial platelet function testing guided de-escalation of antiplatelet treatment was non-inferior to standard treatment with prasugrel at 1 year after (net clinical benefit) [14]. In our study, the rate of periprocedural escalate switch was about 2%. However, in top centers it was six times higher. The reason for significant differences in escalate switching rate between centers is not clear. In some networks old approach with a high rate of prehospital clopidogrel administration may be the reason (despite lack of contraindications for ticagrelor to be administered on top of clopidogrel) [15]. In top 10 centers in Poland (according to newer generation P2Y_12_ inhibitors usage rate), the rate of prehospital clopidogrel administration was lower than in general cohort. This is along to concept proposed in 2011 by experts in Poland to postpone the decision of P2Y_12_ inhibitor administration until cathlab hospital admission [16]. It should be underlined that when this concept was born, ticagrelor and prasugrel were available only in cathlab hospitals. Before introducing of newer generation oral P2Y_12_ inhibitors, clopidogrel was available and widely used in many networks in ambulance [17]. Currently, ticagrelor is also available in growing number of ambulances based on new regulations. So in top 10 centers we observed less clopidogrel administered early and more ticagrelor administered both early as well as during procedure. Also, the periprocedural escalate switching in those centers was about 12% showing that antiplatelet treatment strategy was reassessed during the procedure. Data on the effectiveness of the strategy of early (prehospital) P2Y_12_ inhibitors administration in STEMI patients is not clear. ATLANTIC trial has shown no clear benefit of early ticagrelor administration but the time from loading dose administration to the procedure was relatively short [8]. On the other hand, in many European centers such approach is a part of daily practice in STEMI treatment showing some clinical benefit [9]. All those data on type, time of administration and eventual switch between P2Y_12_ suggest the need for tailored rather than systematic approach, what will be the future challenge in the treatment of patients with ACS.

### Limitations

The main limitation of presented analysis is lack of data on discharge and follow-up antiplatelet treatment as well as in-hospital and follow-up adverse events rate. Only periprocedural events rate was available and we decide not to include it into presented analysis because we wanted to analyze treatment patterns but not impact of selected P2Y_12_ inhibitors on outcomes. We were not able to fully analyze adherence to guidelines due to lack of full data concerning contraindications to a particular P2Y_12_ inhibitor. The analysis is based on national registry data not individually monitored.

## Conclusions

Clopidogrel remains the most common P2Y_12_ inhibitor administered in STEMI patients referred to primary PCI in Poland. The penetration of newer generation P2Y_12_ inhibitors is low, and there are significant differences between networks in the proportion of ticagrelor/prasugrel and clopidogrel usage. These suggest the need for a greater effort to improve adherence to guidelines regarding oral antiplatelet treatment in STEMI.
